# Supplementary material for: Hyposalivation, oral health, and Candida colonization in independent dentate elders
Source: PLoS One. 2020 Nov 25;15(11):e0242832. doi: 10.1371/journal.pone.0242832 (PMC7688165; doi:10.1371/journal.pone.0242832)
Supplement: Attachment — Submitted filename: response to reviewers.docx [file pone.0242832.s002.docx]

*Dear editor,*

*We sincerely thank the reviewers and editor for useful comments and suggestions that helped to improve our manuscript. The revised texts are highlighted in yellow in the manuscript and our responses to the comments are listed in blue italic fonts below in a point-by-point fashion. We hope that the revision satisfies the requirements of the journal and the editor and that the manuscript is now in a suitable form for publication in PLOS One.*

**Point-by-point responses to reviewers and editor’s comments**:

**Journal Requirements**:

1. Please ensure that your manuscript meets PLOS ONE's style requirements, including those for file naming. The PLOS ONE style templates can be found at https://journals.plos.org/plosone/s/file?id=wjVg/PLOSOne_formatting_sample_main_body.pdf and https://journals.plos.org/plosone/s/file?id=ba62/PLOSOne_formatting_sample_title_authors_affiliations.pdf

*Response:*- *We have prepared the revised manuscript according to the instructions provided.*

2. We note that you have included the phrase “data not shown” in your manuscript. Unfortunately, this does not meet our data sharing requirements. PLOS does not permit references to inaccessible data. We require that authors provide all relevant data within the paper, Supporting Information files, or in an acceptable, public repository. Please add a citation to support this phrase or upload the data that corresponds with these findings to a stable repository (such as Figshare or Dryad) and provide and URLs, DOIs, or accession numbers that may be used to access these data. Or, if the data are not a core part of the research being presented in your study, we ask that you remove the phrase that refers to these data.

*Response:*- *We have added the data into the manuscript (Table 6) and deleted the phrase “data not shown”.* All data are provided in the supplementary data file.

Additional Editor Comments:

Thank you for submitting the work to PLoS ONE. While the reviewers and editor see great potential of the study, there are several issues needed to be addressed:

1. In the introduction: The authors mentioned the changes of salivary proteomes based on changes in saliva flow. There is not sufficient discussion of which proteins that may be insufficient or more prevalent that would alter oral candidiasis. There are also a role of denture wearing and candidal species needed to be discussed. The following studies may need to be reviewed and added to the introduction. There was also an interaction between different candidal species associated with denture stomatitis and denture wearing. For example, the *C. glabrata* species seems to associate with *C. albicans* based on proteomic study and there is a reduction in protective proteins in saliva. Please add more of your thought and hypothesis in the introduction based on current literature (see below).

*Response:*- *We thank the editor for the useful suggestions. We have included additional information together with necessary references on the changes in salivary proteome, Candida species interaction, and denture wearing in the introduction and/or discussion as suggested. (Page 4, line 74-77; Page 5, line 93-94; Page 28, line 432-439; Page 29, line 446-453)*

2. In the Methods, please add a table for detailed inclusion and exclusion criteria. The presence of denture should be addressed with denture hygiene and habit, e.g. night denture wearing as suggested by Reviewer #2. Have you documented the denture wearing and hygiene habit? Do you have data on edentulous patients or how many teeth remaining? Please add these data.

*Response:- We have now added a table describing the inclusion and exclusion criteria (Table 1 page 6), and information on denture plaque index (page 9 line 169-177, and Table 6 Page 21) and denture hygiene practice (page 20, line 314-321). Because we aimed to focus on only dentate subjects in this study, there was no edentulous patients.* *We have also added the information on* *number of remaining teeth in Table 2 (page 13).*

3. In the Discussion, please discuss the interaction between the most common non albican species, *C. glabrata* with *C. albicans* and see if this ties back into your introduction. Please also discuss the denture hygiene with oral candidiasis.

*Response:- We have added further discussion on the interaction between C.glabrata and C.albicans and denture hygiene as suggested. (Page 28, line 432-439; Page 29, line 446-453; and Page 20, line 314-321)*

Reviewers' comments:

Reviewer's Responses to Questions

**Comments to the Author**

5. Review Comments to the Author

Please use the space provided to explain your answers to the questions above. You may also include additional comments for the author, including concerns about dual publication, research ethics, or publication ethics. (Please upload your review as an attachment if it exceeds 20,000 characters)

Reviewer #1: I found it interesting that you chose to exclude subjects with oral candidiasis, as this is an important potential outcome of oral Candida species colonization. Overall, a well set-out cross sectional study. The data appears to support the conclusions.

*Response:*- *We thank the reviewer for the positive comments. The reason that we exclude patients with oral candidiasis was because, in this study, we focused on the relationship between hyposalivation, oral health status, and Candida colonization in relatively healthy independent elders. In addition, we aimed to examine Candida carriage in the absence of oral infection. Thus, we did not include patients with oral candidiasis also because it may have resulted from systemic immunocompromising conditions.*

Reviewer #2: Methods should include a description of (1) how denture wearers care for their dentures and (2) any treatment by the study participants for hyposalivation (Biotene). A major gap in the study is the lack of information regarding oral care administered by the study participants.

*Response:*- *We thank the reviewer for the useful suggestions. We have now added the information on denture hygiene practice of denture wearers (page 20, line 314-321) and denture plaque index (Table 6). To ease their dry mouth symptoms, most of the subjects with xerostomia in this study take frequent sips of water (page 12, line 232-234). Furthermore, Biotene is not available in Thailand.*

*Should you have any further questions or suggestions, please contact me at* [*Oranart.m@chula.ac.th*](mailto:Oranart.m@chula.ac.th)*.*

*Best regards,*

*Oranart Matangkasombut*

*Orapin Komin*
